# Osmotically Sensitive TREK Channels in Rat Articular Chondrocytes: Expression and Functional Role

**DOI:** 10.3390/ijms25147848

**Published:** 2024-07-18

**Authors:** Arturo Ponce, Alejandro Ogazon del Toro, Lidia Jimenez, Maria Luisa Roldan, Liora Shoshani

**Affiliations:** Department of Physiology, Biophysics and Neurosciences, Center for Research and Advanced Studies of the National Polytechnic Institute (CINVESTAV-IPN), Ciudad de México 07360, Mexico; alejandro.ogazon@cinvestav.mx (A.O.d.T.); lidia.jimenez@cinvestav.mx (L.J.); luisa.roldan@cinvestav.mx (M.L.R.); shoshani@cinvestav.mx (L.S.)

**Keywords:** articular cartilage, mechanosensitive channels, two-pore K channels, osteoarthrosis, osmosensitive channels

## Abstract

Articular chondrocytes are the primary cells responsible for maintaining the integrity and functionality of articular cartilage, which is essential for smooth joint movement. A key aspect of their role involves mechanosensitive ion channels, which allow chondrocytes to detect and respond to mechanical forces encountered during joint activity; nonetheless, the variety of mechanosensitive ion channels involved in this process has not been fully resolved so far. Because some members of the two-pore domain potassium (K2P) channel family have been described as mechanosensors in other cell types, in this study, we investigate whether articular chondrocytes express such channels. RT-PCR analysis reveals the presence of TREK-1 and TREK-2 channels in these cells. Subsequent protein expression assessments, including Western blotting and immunohistochemistry, confirm the presence of TREK-1 in articular cartilage samples. Furthermore, whole-cell patch clamp assays demonstrate that freshly isolated chondrocytes exhibit currents attributable to TREK-1 channels, as evidenced by activation by arachidonic acid (AA) and ml335 and further inhibition by spadin. Additionally, exposure to hypo-osmolar shock activates currents, which can be attributed to the presence of TREK-1 channels, as indicated by their inhibition with spadin. Therefore, these findings highlight the expression of TREK channels in rat articular chondrocytes and suggest their potential involvement in regulating the integrity of cartilage extracellular matrix.

## 1. Introduction

Articular cartilage (AC) is an aneural and avascular type of connective tissue that covers the articulating surfaces of bones of diarthrodial joints, responsible for various types of movement, including bending, straightening, rotation, and gliding. AC is primarily composed of water and an extracellular matrix (ECM) consisting of collagen fibers and proteoglycans. This composition renders it highly resilient and pliant, providing a smooth, nearly frictionless surface that efficiently supports and distributes forces generated during joint loading and motion [1]. Despite its resilience, articular cartilage is prone to dysfunction following acute injury or chronic diseases such as osteoarthritis (OA), the most common joint disorder [2], largely due to its limited intrinsic repair and regenerative capacity [3,4]. 

Articular chondrocytes (ACHS) are the sole cellular component of articular cartilage, crucial for synthesizing the extracellular matrix (ECM) and maintaining the cartilage’s load-bearing and joint functions [5]. Derived from mesenchymal cells, ACHS are distributed throughout the cartilage, with distinct differentiation from the superficial to the deep zones [6]. ACHS are surrounded by a peripheral extracellular matrix (PEM) layer, forming functional units called chondrons. ACHS inhabit a specialized milieu where mechanical forces, including compression, tension, and shear stress, act upon articular cartilage during activities such as walking, running, and other joint movements [7]. These forces induce alterations in matrix hydration, resulting in variations in the osmolarity and ionic composition of the extracellular environment [8,9].

ACHS are critical for the generation and maintenance of articular cartilage [10,11]. They respond to mechanical forces by regulating the synthesis and degradation of extracellular matrix (ECM) components, such as proteoglycans and collagen, which are essential for the integrity and function of the tissue [12]. Numerous studies have demonstrated that dynamic compression-induced membrane strain elicits responses in chondrocytes aimed at preserving articular cartilage health [13]; for instance, mechanical stimulation of controlled magnitude and frequency applied to chondrocytes in culture induces several physiological responses, including alterations in energy metabolism [14], enhanced ACHS proliferation, and increased ECM component production [15,16,17]. Also, it has been observed that mechanical stimulation promotes the differentiation of mesenchymal stem cells into chondrocytes [18,19]. Conversely, static and high-magnitude compression leads to cartilage degeneration [20,21,22], indicating that the pathogenesis of osteoarthritis (OA) is closely linked to excessive mechanical loading of articular cartilage [23,24,25]. Given these facts, a crucial point of interest regarding the cellular and molecular physiology of chondrocytes is to identify the components and mechanisms involved in their mechanosensitivity and to understand how these are regulated in both health and disease. Nonetheless, so far, this knowledge is still limited. Previous studies have identified many cellular structures and molecules that mediate chondrocyte responses to force, including primary cilia, mitochondria, ion channel proteins, transcription factors, inflammatory cytokines, and microRNAs [20].

Among these diverse components, ion channels stand out due to their significant role as mechanosensors across a wide variety of animal cell types, including endothelial cells, smooth and skeletal muscle cells, hair cells, and some types of neurons, playing various physiologically significant roles, including pressure perception, osmoregulation [26], and blood pressure regulation [27], among many others.

A notable variety of mechanosensitive channels has been described so far, belonging to distinct superfamilies of ion channels (reviewed in [28]), including (1) the large conductance, calcium- and voltage-activated potassium channels (BK) [29,30], (2) some members of the Degenerin/epithelial Na+ channel (DEG/ENaC) superfamily, including Epithelial Sodium Channel (ENaC), acid-sensitive channels (ASICs), and nematode’s Degenerin (DEG) [31,32], and (3) the superfamily of transient receptor potential superfamily of cation channels (TRP). There is evidence that members of the TRPC, TRPV, TRPM, TRPA, and TRPP subfamilies have mechanical or osmotically sensitive properties [33,34]. Among them, particularly notable have been TRPV1, TRPV2, and TRPV4, which belong to the TRPV subfamily [35]. Additionally, (4) the piezo channels (Piezo1 and Piezo2) [36,37,38], (5) some members of the two-pore domain potassium channel family (K2P) [39], including TREK-1, TREK-2, and TRAAK [40,41,42], (6) mechanosensitive channels of the TMEM16/Anoctamin superfamily, a class of Ca^2+^-activated Cl^−^ channels associated with swelling-activated chloride currents (ICl-swell) [43], and (7) some members of the transmembrane 63 (TMEM63/OSCA) superfamily of proteins, originally identified as homologs of the osmotically sensitive calcium-permeable (OSCA) channels in plants [44].

A noticeable great diversity of ion channels has been described to be expressed by articular chondrocytes [45,46], encompassing voltage-gated Na^+^ channels [47], voltage-gated K^+^ channels [48,49,50,51,52], calcium-dependent K^+^ channels [53], voltage-gated calcium channels [54], TRPV channels [55], aquaporins [56], and voltage-gated Cl^−^ channels [57]. Some of them have been shown to participate in the mechanosensitive behavior of AC (reviewed in [58,59,60]), including piezo 1 and 2 [61], TRPV4 [52,62], and swelling-activated chloride channels (TMEM16) [63].

Regarding the expression of mechanosensitive K2P channels in ACHS, it has been recently described that human chondrocytes in primary cultures exhibit immunoreactivity to anti-TREK-1 antibodies [64], suggesting the presence of this type of channel in these cells. However, this study did not investigate other mechanosensitive channels of the K2P family nor explored their functional properties and osmosensitivity.

Therefore, in this study, we used molecular, biochemical, pharmacological, and biophysical techniques to determine whether rat articular chondrocytes express mechanosensitive K2P channels (TREK-1, TREK-2, or TRAAK) and if these channels exhibit mechanosensitive properties like those seen in other cell types, potentially implicating them in the mechanosensitivity of articular chondrocytes. Specifically, we examined whether ion currents associated with these channels respond to changes in osmolarity. As demonstrated below, we found that rat articular chondrocytes express TREK-1 channels, which may play a role in their response to hypo-osmotic changes.

## 2. Results

### 2.1. Expression of TREK-1 (Variant 2) and TREK-2 in Rat Articular Cartilage Is Evidenced by RT-PCR Assays

**Figure 1:**
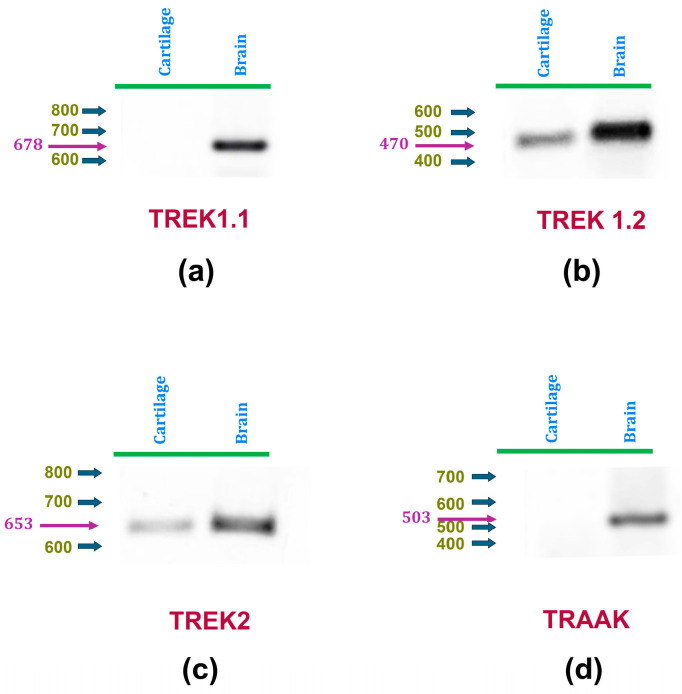
mRNA expression of K2P channels TREK-1 and TREK-2 in articular chondrocytes is detected by RT-PCR assays. Panels (**a**,**b**) display representative RT-PCR assay images using cDNA derived from rat articular cartilage (left) and brain (right) as templates. Specific primers were used to amplify cDNA sequences of (**a**) TREK1 variant 1, (**b**) TREK1 variant 2, (**c**) TREK2, and (**d**) TRAAK. In all panels, blue arrows indicate the positions of molecular weight markers, while magenta arrows and numbers denote the expected weights.

As an initial attempt to investigate the expression of mechanosensitive K2P channels in rat articular cartilage, RT-PCR assays were performed to determine the presence of TREK-1, TREK-2, and TRAAK in this tissue. To this end, as described in Table 1, specific primers were designed for each channel type using their respective RefSeq accession numbers with NCBI’s Primer-BLAST tool [65]. Because the TREK-1 gene (*Kcnk2*) has been described to produce two splicing variants [66,67], distinct primers were created for each variant (TREK-1.1 and TREK-1.2). RT-PCR assays were conducted on cDNA extracted from articular cartilage obtained from hip samples, and on rat brain samples as positive controls. As shown in Figure 1, TREK-1.1 was found to be expressed in the brain but not in cartilage, whereas TREK-1.2 is expressed in both tissues. TRAAK expression is only found in rat brains, while TREK-2 expression was detected in both rat cartilage and brain. These results indicate, therefore, that rat articular chondrocytes express TREK-1.2 and TREK-2, but not TREK-1.1 nor TRAAK.

**Table 1:** Primer properties.

Channel	NCBI RefSeq	Product	Primer	Primer Sequence	Primer
Name	Acces. Num.	Size	Sense		Size
TREK-1.1	NM_172041.2	678	Forward	GAAAAGGAGCGTCTACCTGG	20
			Reverse	AGGACACAGCCAAACAGGATG	21
TREK-1.2	NM_172042.2	470	Forward	CCGGCTATACCGCAGGAGTG	20
			Reverse	ACCTTCAGTTCGTGGGGAGAT	21
TREK-2	NM_023096.2	653	Forward	GGTGCAAACACCCAACCAAG	20
			Reverse	GAGTTTCCTACCGGGCTGAC	20
TRAAK	NM_053804.3	503	Forward	CCTGGAAGGTTGGACTCTGC	20
			Reverse	ATTGCCGTAGCCGATGGTAG	20

### 2.2. The Expression of TREK-1 Proteins in Rat Articular Cartilage Is Demonstrated by Western Blot and Immunohistochemistry Procedures

Next, we aimed to demonstrate the expression of TREK-1 channels in rat articular cartilage via recognition by a specific antibody against TREK-1 (αTREK-1) using Western blot and immunostaining techniques on hip cartilage slices.

**Figure 2:**
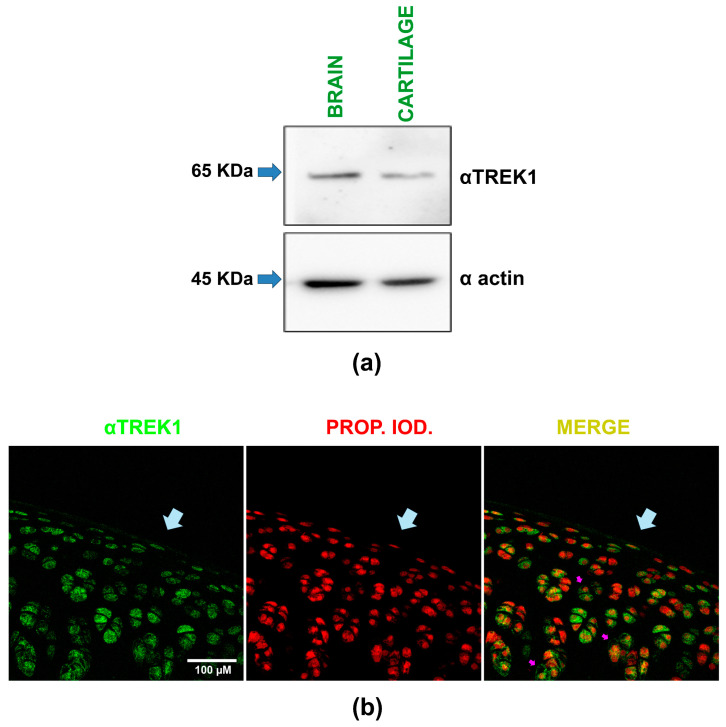
TREK-1 protein expression in rat articular cartilage is demonstrated by Western blot and immunofluorescence assays. (**a**) Representative image of Western blot assays performed on rat brain and articular cartilage homogenates. The upper section shows results from the membrane probed with an anti-TREK-1 antibody (αTREK-1), while the lower section displays results from the membrane probed with an anti-actin antibody (α actin). Arrows indicate the expected molecular weight (KDa). (**b**) A representative composite image, captured via confocal microscopy, illustrates immunofluorescence assays on slices of rat hip cartilage. A primary antibody against TREK-1 (αTREK1) was used, followed by fluorescein detection, with nuclei counterstained using propidium iodide. The images show the same field with separate channels displaying TREK-1 expression in green (left), nuclei in red (middle), and the merged view (right). Light blue arrows highlight the cartilage surface, while violet arrows indicate a peripheral, dotted distribution around the nucleus of specific chondrocytes.

By Western blot assays, we compared TREK-1 expression in rat cartilage and brain samples, with the brain serving as a positive control. As shown in Figure 2a, these assays revealed a band with a molecular weight of about 65 KDa in both brain and cartilage lysates when probed with the αTREK-1 antibody. These results show, therefore, that rat articular cartilage expresses TREK-1 proteins.

This conclusion is further supported by the results obtained from immunofluorescence assays performed on hip cartilage slices. As depicted in the composite image in Figure 2b, incubating these samples with the same specific antibody against TREK-1 and subsequent staining with fluorescein reveals immunoreactive marks on the chondrocytes within the cartilage sample. In Figure 2b, the green staining indicates TREK-1 protein expression, while the red one signifies propidium iodide counterstaining, highlighting the chondrocyte nuclei. The pink arrows highlight chondrocytes where TREK-1 is expressed peripherally and in a dotted pattern, present in most chondrocytes regardless of their location in the cartilage. Thus, the results of both experimental approaches (Western blot assays and immunohistochemistry) confirm that TREK-1 proteins are expressed in rat articular cartilage.

### 2.3. Freshly Isolated Chondrocytes Exhibit Ionic Currents Associated with the Presence of TREK (1/2) Channels

Furthermore, we performed whole-cell patch clamp experiments on freshly isolated chondrocytes to search for ion currents that could be attributable to the expression of TREK-1 and/or TREK-2 channels in rat articular chondrocytes.

To eliminate the ion currents associated with voltage-gated K^+^ channels, as previously described [48], the extracellular recording solutions were supplemented with 10 mM TEA and 5 mM 4AP. Likewise, to exclude swelling-activated chloride currents [63], Cl^−^ was replaced by gluconate- (Gln-), an impermeant anion, in both intra- and extra-cellular solutions. The composition of recording solutions is described in Table 2.

#### 2.3.1. Ion Currents Recorded from Freshly Dissociated Chondrocytes Are Induced by TREK Channel Activators Arachidonic Acid and ml335

To determine whether chondrocytes express ion currents associated with TREK-1 and/or TREK-2 channels, we examined the effects of two compounds known to activate these channels in various cell types: arachidonic acid (AA) [39,68] and ml335. These compounds selectively activate TREK-1 and TREK-2 over TRAAK channels by modulating the C-type selectivity filter through binding to the K2P modulator pocket [69,70].

We used two distinct current recording protocols to examine the effects. In the first protocol, we continuously recorded the membrane ion current while holding a constant voltage of 0 mV, changing the extracellular medium by perfusion. Starting with a control condition (without treatment), we added the test compound at increasing concentrations of 1, 5, and 10 μM for 30 s each, followed by a washout. For statistical analysis, we measured the membrane capacitance of each cell as an estimator of the cell’s membrane surface to express the results as current density (pA/pF). In the second protocol, we recorded membrane currents in response to a series of square voltage pulses ranging from −160 to 80 mV in +20 mV increments, starting from a holding potential (Vh) of −80 mV. Ion currents were recorded before (control), during, and after (washout) the application of AA or ml335 to the extracellular medium.

**Figure 3:**
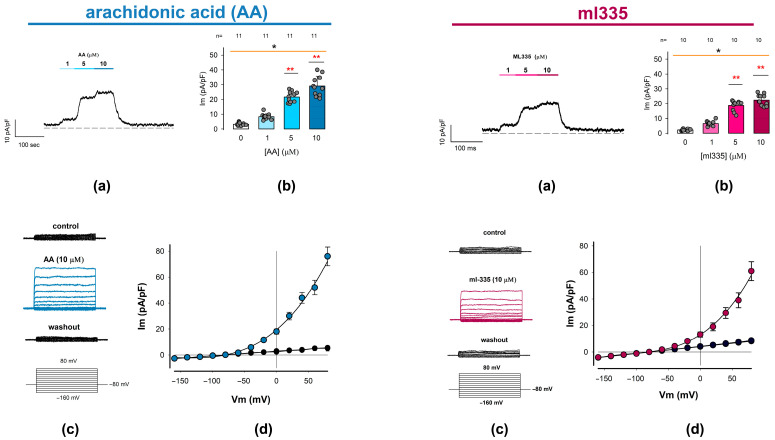
Outwardly rectifying currents are induced in freshly dissociated chondrocytes by adding TREK channel activators to the external medium. Whole-cell patch clamp experiments were conducted to examine the effects of arachidonic acid (AA) and ml335, both known activators of TREK channels (1 and 2), on membrane ion current recordings. The figure comprises two sets of panels: the (**Left**) set shows the effect of AA, while the (**Right**) set shows the effect of ml335. On each set, (**a**) show representative ion current traces continuously recorded while clamping the membrane voltage at 0 mV, before, during the addition of either AA or ml335 at increasing concentrations to the medium, and after washout, as indicated by the bars above the traces. (**b**) Bar charts compare the mean value (±S.E.) of ion current density measured at each activator concentration, as indicated below the bars. The average value is derived from the number of trials (n) indicated above the bars. An asterisk above either orange line denotes a statistically significant difference (*p* < 0.01) from an ANOVA test, while red asterisks (**) above bars indicate a significant statistical difference between the mean value of the given treatment concentration and the corresponding control (0 µM) group. (**c**) Displays three series of ion currents recorded before (upper), upon addition of either AA or ml335 (middle) to the external medium, and after washing out (lower). Ion current episodes were induced by a series of step voltage pulses ranging from −100 to +80 mV from a holding potential of −80 mV. (**d**) I-V plots comparing the relationship between the mean value (±S.E.) of ion current density (pA/pF) and the testing voltage (mV) for AA and ml335 and their respective controls. Colored symbols (blue/red) correspond to treatment I-V plots (AA/ml355).

Figure 3 summarizes the findings for AA and ml335 in two sets of panels. The upper panels depict (a) a representative trace of ion current continuously recorded while changing the extracellular media and (b) the statistical analysis of the response elicited by the first stimulation protocol. The lower panels show (c) a series of representative current traces elicited by a series of square-shaped voltage pulses, recorded before, during, and after the presence of either activator compound, and (d) the corresponding current density–voltage (I-V) relationships.

As illustrated in panel (a) of each compound set, the addition of the activators to the external medium results in a positive shift in the current magnitude. This effect was detectable even at the lowest tested concentration (1 µM) and progressively increased with higher activator concentrations (5 and 10 µM). Moreover, the observed increase is reversible, as evidenced by the decrease in the level of current density upon washout, returning to levels comparable to baseline. The average values (±S.E.) of the current density obtained from a number (n) of repeats are the following: for AA, 0 μM (3.3 ± 0.3 pA/pF, n = 11), 1 μM (8.3 ± 0.6 pA/pF, n = 11), 5 μM (21.7 ± 1.1 pA/pF, n = 11), and 10 μM (29.1 ± 2.0 pA/pF, n = 11); while, for ml335, 0 μM (2.2 ± 0.2 pA/pF, n = 10), 1 μM (6.6 ± 0.5 pA/pF, n = 10), 5 μM (18.7 ± 1.0 pA/pF, n = 10), and 10 μM (22.5 ± 1.1 pA/pF, n = 10). As shown in panel (b), the statistical analysis of these data indicates that both activators (AA and ml335) produce a statistically significant effect on the current density of chondrocytes (*p* < 0.001, Kruskal–Wallis). Also, in both cases, the effect is significantly greater than the control from 5 μM (*p* < 0.001, Dunn test).

On the other hand, as can be seen in the lower panels (c and d), stimulation with square pulses of voltage in the absence of AA or ml335 (control) does not induce noticeable currents; however, in the presence of either of the two activators, the same protocol of stimulation induces ion currents exhibiting no voltage dependence, whose steady state magnitude increases with depolarizing voltage steps. As shown in panel (d), the I-V profile of these currents displays prominent outward rectification and has an inversion potential around the theoretical equilibrium potential for K^+^ under these experimental conditions (−80 mV).

These biophysical characteristics are similar to those described for currents produced by TREK-1 and TREK-2 channels [71]. Consequently, the results obtained with both compounds AA and ml335 allow us to confidently state that the currents recorded evoked by any of these compounds are due to the presence of TREK channels in the membrane of rat articular chondrocytes; therefore, from now on, we will refer to these currents as ITREK.

#### 2.3.2. ITREK Currents Are Notoriously Inhibited by Spadin, a Specific Inhibitor of TREK-1 Channels

Given that neither AA nor ml335 are selective for TREK-1 or TREK-2, we cannot determine the relative contribution of these two channel types to ITREK. Nonetheless, various inhibitors of K2P channels are known to be more specific than activators. Among these inhibitors, spadin, a peptide, has been reported to selectively inhibit TREK-1 over TREK-2 or other K2P channels [70,72,73]. Therefore, we conducted whole-cell patch clamp trials to test the effect of spadin on ion currents previously activated by either AA or ml335 in freshly dissociated articular chondrocytes (ITREK).

**Figure 4:**
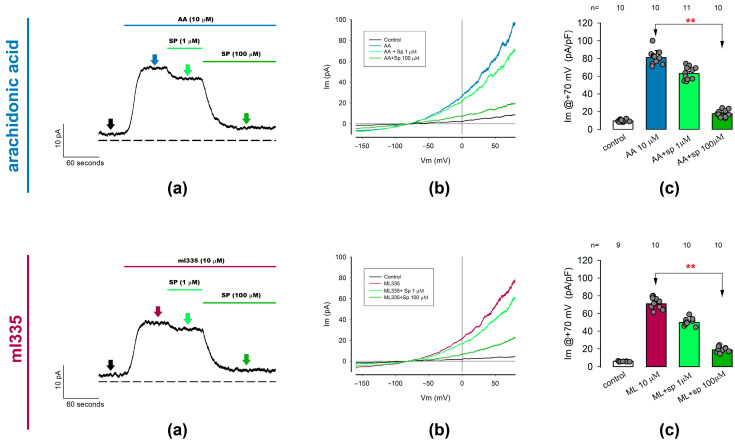
Outwardly rectifying currents induced by TREK channel activators (AA and ml335) are suppressed by spadin, a specific TREK1 channel blocker. Whole-cell patch clamp assays were performed to investigate the effect of spadin on currents previously activated by arachidonic acid (10 µM) or ml335 (10 µM) in freshly dissociated chondrocytes. The figure consists of two rows of three panels each. The (**Upper** row) illustrates the effect of spadin on AA-activated currents, while the (**Lower** row) shows its effect on ml335-activated currents. (**a**) Displays representative ion current traces recorded continuously with the membrane voltage held at 0 mV. Currents were initially activated by either AA or ml335, followed by the addition of spadin at increasing concentrations of 1 and 100 µM. The dotted line represents the zero level for membrane current density. Arrows indicate the time ramp stimulation was made to sample currents for statistical purposes, colors are intended to match the different treatments. (**b**) Presents representative sets of traces of ion currents induced by ramp-shaped voltage pulses ranging from −150 to +80 mV, recorded before (control), 30 seconds after the addition of activators (AA, upper row) or ml335 (lower row), and 30 seconds after the addition of spadin at concentrations of 1 and 100 µM, as indicated in the inset. (**c**) Depicts bar charts comparing the mean value (±S.E.) of the ion current density measured at +70 mV for each treatment condition. The treatment conditions are indicated below the bars. Red asterisks (**) denote significant statistical differences (*p* < 0.001) in the mean values of the bars indicated by arrows, determined by a Dunn test.

As shown in Figure 4, we tested the effect of spadin at two different concentrations (1 μM and 100 μM) on currents previously activated by AA or ml335 at 10 μM. In both cases, we made continuous recordings of ion current while perfusing the activator (either AA or ml335) to the external medium, followed by spadin in concentrations from the lowest to highest (as illustrated in panels (a) of each set in Figure 4). Then, to evaluate the statistical significance of the effect, we recorded, in each experimental condition, the density of currents produced by a ramp-shaped voltage stimulation from −150 to +100 mV. Panels (b) show representative traces of ionic current recorded in response to a voltage stimulation in the form of a ramp under the different experimental conditions described in the inset and at times indicated by the arrows of the corresponding color on panels (a). Furthermore, for comparative purposes, we considered the value of the current density at +70 mV in all chondrocytes and experimental conditions. As seen in the (c) panels, statistical analysis of the current density values indicates a significant reduction in the mean value of the current density previously activated by either AA or ml335 value because of treatment with 100 μM spadin but not with 1 μM.

Thus, these results lead us to assert that ITREK is the result of a significantly important contribution of TREK-1-type channels in the membrane of rat articular chondrocytes.

### 2.4. Hypo-Osmolar Shock Induces Activation of Ion Currents Attributable to TREK-1 Channels

Next, we performed whole-cell patch clamp tests to find out if the ionic currents due to TREK-1 channels are osmotically sensitive. For this purpose, we performed tests in which we continuously recorded the membrane current in response to a voltage protocol consisting of the alternating stimulation of square pulses of +70 and −70 mV from a Vh of 0 mV. Thus, the membrane current was recorded continuously, starting with an iso-osmolar solution (330 mOsm), and after the recorded cell was challenged by perfusion with hypo-osmotic solutions of 280 mOsm and 160 mOsm and further returned to the former condition.

**Figure 5:**
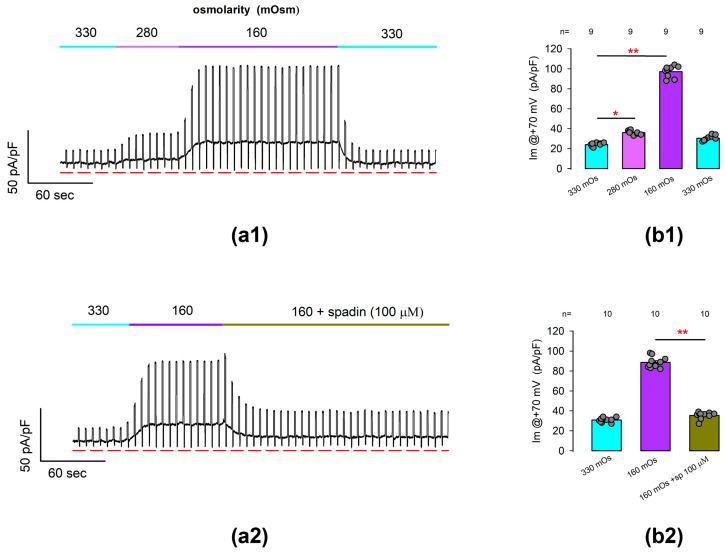
Outwardly rectifying currents induced by hypo-osmotic challenges are suppressed by treatment with spadin. (**a1**) shows a representative example of continuous recording of ionic current resulting from repetitive stimulation in which square pulses of voltage at +60 and −60 with a duration of 200 ms were alternately given every 5 s from a holding voltage of 0 mV. As indicated in the top bar of each stroke, the recordings were initially made with an iso-osmolar external medium (360 mOSm), and later, the medium was replaced by hypo-osmolar solutions of 280 and 160 mOsm sequentially. The red lines below the strokes indicate the value of 0 for the current density. (**a2**) shows a representative trace like that of (**a1**), but in which the cell was first challenged with hypo-osmolar media (160 mOsm) and then perfused with 100 μM spadin even in the presence of the hypo-osmolar medium. (**b1**,**b2**) are bar charts comparing the average (±S.E.) value of current density, measured at +60 mV at each treatment condition indicated below bars. The numbers above each bar indicate the number of repetitions. The circles above each bar represent the individual measurements for each case. (*) and (**) indicates a statistically significant difference of *p* < 0.01 and *p* < 0.001, correspondingly, between the bars separated by the lines, determined by a *t*-test.

Figure 5(a1) shows a representative record of these trials. As can be seen, the substitution of the extracellular medium by hypo-osmolar solutions of 280 and 160 produced an increase in the basal current density value, obtained when the voltage is set at its basal level, but there is also an increase in the magnitude of the current obtained by stimulating the membrane at +70 mV.

Statistical comparison of the average values of current density at +70 mV indicates, as illustrated in Figure 5(b1), that hypo-osmotic challenges induce statistically significant changes in the density of ionic currents recorded from freshly dissociated articular chondrocytes under experimental conditions like those used in previous tests, and which could therefore be attributed to TREK channels (ITREK).

To corroborate this hypothesis, we conducted similar assays to test whether the spadin was able to inhibit the currents induced by hypo-osmolar challenges. Figure 5(a2) shows a representative record of the current obtained from a chondrocyte stimulated in the same way described above and in which the increase in current was initially evoked when changing the isotonic medium to hypo-osmotic (160 mOsm). As can be seen, the addition of spadin (100 µm) to the hypo-osmotic medium caused the magnitude of this current to decrease, even in the presence of the hypo-osmotic medium.

As shown in Figure 5(b2), the average (±S.E.) current density at +70 mV (Im@ + 70 mV) as measured from cells bathed into a hypo-osmolar solution (88.6 ± 1.7 pA/pF, n = 10) is significantly higher (*p* < 0.001, *t*-test) than that of cells bathed into an iso-osmolar solution (30.8 ± 0.7 pA/pF, n = 10). Subsequently, the addition of spadin to the hypo-osmolar medium caused a significant decrease (*p* < 0.001, *t*-test) in the average value of Im@ + 70 mV (35.4 ± 1.1 pA/pF, n = 10).

Therefore, these results suggest that the hypo-osmotically induced enhancement of ion currents in freshly dissociated rat articular chondrocytes is mostly due to TREK-1 channels and, to a negligible part, TREK-2 channels or other types of channels.

## 3. Discussion

An important research objective in ACHS’s cellular and molecular physiology is identifying the variety of ion channels these cells express. Furthermore, identifying those ion channels that act as mechanosensors is particularly crucial as the first step to recognizing the signaling pathways that enable chondrocytes to adjust their environment in response to mechanical stimuli [74]. This goal is essential, as a deeper understanding of the variety of chondrocytes’ mechanosensitive ion channels could provide valuable insights into the causes of pathological conditions, including the significant condition of articular osteoarthritis [75].

This study aimed to investigate the expression and functional role of mechanosensitive two-pore domain potassium channels (K2Ps), specifically TRAAK, TREK-1, and TREK-2, in rat articular chondrocytes. Our findings provide significant insights into the presence and potential physiological relevance of these channels in articular cartilage.

RT-PCR assays indicated the presence of TREK-1 (specifically the TREK-1.2 variant) and TREK-2 mRNA in rat articular cartilage, while TREK-1.1 and TRAAK were not expressed in this tissue. These results were corroborated by Western blot and immunohistochemistry analyses, which confirmed the presence of TREK-1 proteins in articular cartilage. These results support the notion that TREK-1 and TREK-2 channels could play significant roles in the mechano-transduction processes of chondrocytes.

Whole-cell patch clamp experiments provided functional evidence of TREK channel activity in chondrocytes. We observed that both arachidonic acid (AA) and ml335, known activators of TREK channels, induced outwardly rectifying currents with a characteristic I-V profile. The fact that these currents were significantly suppressed by spadin, a specific inhibitor of TREK-1 channels [73], suggests a predominant contribution of TREK-1 channels in these responses. The reversible nature of the AA-induced currents further supports the functionality of TREK channels in ACHS.

Furthermore, the mechanosensitivity of TREK channels was evaluated by examining their response to osmotic changes. Hypo-osmotic challenging elicited significant increases in membrane current density, which were notably inhibited by spadin. This suggests that TREK-1 channels are not only present but also functionally contribute to the osmotically sensitive behavior of articular chondrocytes. The ability of TREK channels to respond to mechanical stimuli, such as changes in osmolarity, aligns with their established roles in other cell types and reinforces their importance in maintaining chondrocyte physiology.

These results suggest, therefore, that TREK-1 and perhaps to a lesser extent TREK-2 could function as mechanosensors of articular chondrocytes, in addition to other channels that are already known to participate in the mechanosensitivity of these cells [58,59,60], including piezo1 and piezo2 [61], TRPV4 [52,62], and TMEM16 [63].

Also, these findings expand the variety of K2P channels described as being expressed in chondrocytes, including TWIK-1, TWIK-2, and TASK-2 [76,77]. As is known, K2P channels, also called leak channels, contribute significantly to the background currents, which modulate the membrane potential of both excitable and non-excitable cells [78,79]. Because hypo-osmotic changes have previously been shown to produce changes in membrane potential [53], it is also possible that TREK channels act by modulating the membrane potential of chondrocytes in response to changes in membrane osmolarity or mechanical stimuli, an effect that has so far been attributed to potassium channels BK [53].

The results described here, which demonstrate the expression of TREK-1 channels, agree with another recent report showing their expression in primary human chondrocytes [64], as well as in other types of cells related to movement, including human intervertebral disc cells [80] and tendon cells (tenocytes) [81].

### 3.1. Implications for Cartilage Health and Disease

The expression and functional activity of TREK channels in chondrocytes may have important implications for understanding the mechanobiology of articular cartilage. Given the crucial role of mechanical forces in regulating chondrocyte function and maintaining cartilage integrity, TREK channels could be pivotal in translating mechanical stimuli into biochemical signals. This mechano-transduction pathway may influence various cellular processes, including matrix synthesis and degradation, energy metabolism, and cell proliferation [82,83].

Our findings also suggest potential therapeutic targets for conditions like osteoarthritis (OA), where cartilage degeneration is associated with disrupted mechano-transduction [84,85,86]. Modulating TREK channel activity could provide novel strategies for preserving cartilage health and preventing or slowing the progression of OA [75].

### 3.2. Limitations and Future Directions

While our study provides substantial evidence for the presence and function of TREK channels in chondrocytes, several limitations warrant further investigation.

The precise contributions of TREK-2 channels, which were also expressed in articular cartilage, remain to be fully elucidated. Additionally, the in vivo relevance of our findings needs to be confirmed through animal models and clinical studies. Future research should also explore the interactions between TREK channels and other mechanosensitive elements in chondrocytes, such as primary cilia and integrins. Understanding the broader mechano-transduction network will provide a more comprehensive view of how mechanical forces regulate cartilage biology and pathology.

In conclusion, our study demonstrates that TREK-1 and TREK-2 channels are expressed in rat articular chondrocytes and play significant roles in their mechanosensitivity and osmosensitivity. These findings enhance our understanding of chondrocyte mechano-transduction and offer potential avenues for therapeutic intervention in cartilage-related disorders.

## 4. Materials and Methods

### 4.1. RT-PCR Assays

Tissue samples: Rat brains and hip condyles were promptly frozen in liquid nitrogen. Following grinding, total RNA was extracted from the tissue using a Trizol reagent (Invitrogen, Waltham, MA, USA) and quantified via UV spectroscopy. Subsequently, cDNA synthesis was performed using Superscript III reverse transcriptase and oligo (dT)12–18 primer (Invitrogen). PCR assays were conducted utilizing a PCR system 2400 (Perkin Elmer, Waltham, MA, USA). Custom primers were developed for each channel type using NCBI’s Primer-BLAST tool [65], based on the corresponding NCBI’s RefSeq accession number, as described in Table 1.

PCR trials were run with a start temperature of 90 °C for 20 min, then 35 cycles of denaturing for 20 s at 95 °C, followed by alignment at 60 °C for 15 s and extension at 72 °C for 30 s, with a final extension period at 72 °C for 10 min, then kept at 4 °C. Amplified products were resolved by subjecting samples to electrophoresis in agarose gels and stained with propidium iodide.

### 4.2. Western Blot Assays

Brain and articular cartilage samples were chopped and frozen immediately after collection by immersion in liquid nitrogen. Subsequently, frozen samples were ground with mortar and pestle and then lysed with RIPA buffer complemented with PMSF, Sodium orthovanadate and a protease inhibitor cocktail containing a protease inhibitor mix (Cat. sc-24948A. Santa Cruz Biotechnology, Dallas, TX, USA) for 15 min at 4 °C. The lysates were transferred to 1.5 mL microcentrifuge tubes, homogenized using an ultrasonic processor (Cole Parmer GEX 130, Vernon Hills, IL, USA), and centrifuged at 14,000 rpm for 20 min at 4 °C. The supernatant was collected, and the protein concentration was measured using a BCA Protein Assay (Cat. 23228 and 23224. Thermo-Scientific, Waltham, MA, USA). Samples were mixed with 4× loading buffer and heated for 5 min at 90 °C, and proteins (50 μg/lane) were separated on an 8% SDS-PAGE gel at a constant 100 V for 90 min. The gel was transferred to a PVDF membrane (Cat. RPN303F, Amersham Life Science, Slough, Buckinghamshire, UK) using a semi-dry transfer cell (Cat. 1703940, BIORAD, Hercules, CA, USA) at 35 volts for 30 min. The PVDF membrane was blocked with a solution of 5% semi-skimmed milk and 3% bovine serum albumin (BSA) (BAH63, Equitech-Bio, Kerrville, TX, USA) for 1 h at room temperature. Primary antibodies, Rabbit Anti TREK-1 (1:250, Cat. APC-047, Alomone Labs, Jerusalem, Israel) and Mouse anti-Actin (1:1000, provided by Dr. Manuel Hernández, CINVESTAV, Ciudad de México, México), were incubated overnight at 4 °C. This was followed by ten washes with TBS-T (Santa Cruz Biotechnology, Cat. sc-362305) containing 0.01% Tween 20 (Sigma, Cat. P1379, St. Louis, MO, USA). Secondary antibodies (Anti-Rabbit HRP, Cat. 656120, and Anti-Mouse HRP, Cat. 626520, Thermo Fisher Scientific, Waltham, MA, USA) were incubated for 45 min at room temperature, followed by ten washes with TBS-T. The membranes were developed using Immobilon Western (Cat. WBKLS0500, MILLIPORE, Burlington, MA, USA), and the immunoblots were imaged using the FUSION FX6-XT system (WITEC AG, Sursee, Switzerland).

### 4.3. Immunofluorescence of Cartilage Slices

Hip condyles were fixed by immersion in a solution of 4% paraformaldehyde (PFA) in PBS for 48 h at 4 °C, then kept, for cryoprotection sake, in a solution (12% sucrose in Dulbecco’s Phosphate-Buffered Saline, pH 7.3) for 24 h. After that, cartilage slices, 20 μM thickness, were obtained using a cryostat (MC1510, Leica Microsystems GmbH, Wetzlar, Germany), then preserved in a cryo-preservative solution (4% ethylene glycol, 1% polyvinylpyrrolidone in 0.1 M PBS at pH 7.3). Selected slices underwent permeabilization by 1 h incubation with a PBST solution (0.2% Triton X-100 in PBS, pH 7.3), followed by three washes. Blocking was achieved by incubating the slices in a blocking solution (0.05% TX100 + 4% BSA in PBS) for 2 h at room temperature, followed by three washes with PBS. The slices were then incubated with anti-TREK-1 antibody (Alomone, APC-047), diluted 1:200 in blocking solution (0.05% TX100, 4% BSA, 0.1% NaN_3_ in PBS) overnight at 4 °C. The next day, the samples were rinsed three times. Next, they were incubated with Biotin-conjugated Goat Anti-Rabbit IgG (ZYMED^®^, San Francisco, CA, USA), diluted 1:200 in blocking solution for 1 h, followed by three washes. Then, they were stained by 1 h incubation with FITC-Streptavidin (ZYMED^®^) diluted 1:50 in PBS. After three additional washes in PBS, they were counterstained with propidium iodide diluted in PBS. Finally, after six washes, they were mounted on glass slides using a mounting medium (Vectashield VECTOR Labs^®^, Newark, CA, USA) and covered with coverslips. The edges were sealed with nail polish.

#### Confocal Microscopy

Images were captured with a confocal microscope (Leica TG5 SP8; Leica Microsystems GmbH) with a ×63 oil immersion objective and processed with NIH’s Image Processing and Analysis in Java (ImageJ) version 1.53a [87].

### 4.4. Recording of Ion Currents from Freshly Dissociated Chondrocytes

#### 4.4.1. Cell Culture of Freshly Dissociated Articular Chondrocytes

Articular chondrocytes were cultured as described elsewhere [48,63]. Minced hip condyles were exposed to 0.2% type II collagenase in PBS for 2–3 h at 37 °C. After digestion, ACHS were mechanically dissociated by gentle re-pipetting, then washed and centrifuged twice, and then suspended in DMEM supplemented with 10% calf serum. Freshly dissociated ACHS were then seeded at 1–3 × 10^5^ cells/mL onto glass coverslips and maintained under sterile conditions at 37 °C in a humidified atmosphere containing 5% CO_2_ until required.

#### 4.4.2. Whole-Cell Patch Clamp Assays

Ion currents were recorded using the whole-cell patch clamp technique, following established protocols outlined elsewhere [88,89]. Coverslips containing articular chondrocytes were placed in a recording chamber mounted on the stage of an inverted microscope (Diaphot 300, Nikon, Japan). The chamber was filled with an extracellular solution, whose composition is described in Table 2, and kept under continuous perfusion. To establish electrical contact between the bathing solution and the reference electrode, a U-shaped glass tubing containing 2% agarose in 500 mmol/L KCl was utilized. The reference electrode was positioned within a chamber containing KCl 500 mM. Micropipettes, crafted from borosilicate glass tubing (cat. 34500-99, Kimble Chase, Vineland, NJ, USA), were fashioned using a horizontal puller device (P-87, Sutter Instrument Co., Novato, CA, USA) to achieve a tip resistance of 2–5 MΩ. These pipettes were then backfilled with solution IS (Table 2) and connected to a piezoelectric-driven micromanipulator (PCS6000, Burleigh Co., Bismarck, ND, USA) via a pipette holder. The pipettes were guided to the cells, and patch rupture was initiated by suction once the gigaseal exceeded values of 2 GΩ (typically 5 GΩ).

#### 4.4.3. Measurement of Membrane Capacitance

Capacitive transients, elicited by hyperpolarizing square voltage pulses ranging from −100 to −110 mV, were generated and recorded at a frequency of 10 KHz. Subsequently, offline estimation of membrane capacitance was performed by integrating the area under the capacitive transient curve at the onset of the pulse. This integrated value was then divided by the pulse amplitude (−10 mV) using the following formula:

$$\;Cm=\frac{\int_{t0}^{\infty }Ic\cdot ∆t}{∆V}$$

where *Cm* represents membrane capacitance, *Ic* denotes capacitive current, and Δ*V* signifies the pulse amplitude (−10 mV).

The estimate of the integral was carried out using the Clampfit module of pClamp 8.0 (Molecular Devices, San Jose, CA, USA). Cell membrane area was further determined, assuming a specific capacitance of 1 µF/cm^2^ [90].

### 4.5. Solutions

Table 2 describes the composition of the recording solutions used in this work, including extracellular and pipette solutions. The solution named 330 mOsm is the same as used in the initial trials. In the table, the term (gln) is an abbreviation of gluconate, used as an impermeant anion replacing chloride anion.

### 4.6. Analysis of Data

The ionic current values, as the estimation of membrane capacitance, were obtained with the Clampfit module of pClamp 8.0 (Molecular Devices).

Statistical tests were made with EXCEL’s statistical analysis modules (Microsoft 365) and with the analysis tools of Sigmaplot for Windows version 15. The statistical analysis of assays involving more than two groups consisted of performing one-way tests (ANOVA) followed by multiple comparisons versus control. A test for equal variance (Brown–Forsythe) was performed first; when it failed, a nonparametric test (Kruskal–Wallis One-Way Analysis of Variance on Ranks) was used instead, followed by a multiple comparison versus the control group (Dunnet’s method). In a simple two-group comparison, a *t*-test was performed. A minimal value of *p* < 0.01 was considered to reject a NULL hypothesis.

## Figures and Tables

**Table 2:** Composition of recording solutions (mM).

Extracellular Solutions									
Name	NaGln	TEACl	KGln	CaCl_2_	MgCl_2_	Glucose	HEPES	Mannitol	4AP
330 mOsm	52.3	20	5	1.8	1	5	10	150	5
280 mOsm	53.3	20	5	1.8	1	5	10	100	5
180 mOsm	53.3	20	5	1.8	1	5	10	0	5
Intracellular solution									
IS	5	10	115	1	1	0	10	0	0

## Data Availability

Data are contained within the article.
